# Redox-Dependent Structural Modification of Nucleoredoxin Triggers Defense Responses against *Alternaria brassicicola* in *Arabidopsis*

**DOI:** 10.3390/ijms21239196

**Published:** 2020-12-02

**Authors:** Chang Ho Kang, Joung Hun Park, Eun Seon Lee, Seol Ki Paeng, Ho Byoung Chae, Jong Chan Hong, Sang Yeol Lee

**Affiliations:** Division of Applied Life Sciences (BK21+) and Plant Molecular Biology and Biotechnology Research Center, Gyeongsang National University, Jinju 52828, Korea; jacobgnu69@gnu.ac.kr (C.H.K.); jazzc@nate.com (J.H.P.); dmstjsl88@hanmail.net (E.S.L.); skpaeng@gmail.com (S.K.P.); truedaisy@hanmail.net (H.B.C.); jchong@gnu.ac.kr (J.C.H.)

**Keywords:** thioredoxin (TRX) family proteins, fungal pathogen, structural change, plant disease resistance

## Abstract

In plants, thioredoxin (TRX) family proteins participate in various biological processes by regulating the oxidative stress response. However, their role in phytohormone signaling remains largely unknown. In this study, we investigated the functions of TRX proteins in *Arabidopsis thaliana*. Quantitative polymerase chain reaction (qPCR) experiments revealed that the expression of *ARABIDOPSIS NUCLEOREDOXIN 1* (*AtNRX1*) is specifically induced by the application of jasmonic acid (JA) and upon inoculation with a necrotrophic fungal pathogen, *Alternaria brassicicola*. The AtNRX1 protein usually exists as a low molecular weight (LMW) monomer and functions as a reductase, but under oxidative stress AtNRX1 transforms into polymeric forms. However, the AtNRX1M3 mutant protein, harboring four cysteine-to-serine substitutions in the TRX domain, did not show structural modification under oxidative stress. The *Arabidopsis* *atnrx1* null mutant showed greater resistance to *A. brassicicola* than wild-type plants. In addition, plants overexpressing both *AtNRX1* and *AtNRX1M3* were susceptible to *A. brassicicola* infection. Together, these findings suggest that AtNRX1 normally suppresses the expression of defense-responsive genes, as if it were a safety pin, but functions as a molecular sensor through its redox-dependent structural modification to induce disease resistance in plants.

## 1. Introduction

Reactive oxygen species (ROS) generated from diverse internal and external sources play crucial roles in plants, as in human and mammalian systems, especially in the control of plant growth, development, and phytohormone signaling under non-stressed conditions [1,2]. However, exposure to various biotic/abiotic stresses, such as drought, salinity, heat, cold, high light intensity, and pathogen attacks amplifies ROS production [3]. The resulting oxidative stress not only triggers defense signaling to protect the plant host but also causes cellular damage by denaturing important intracellular macromolecules [3,4,5]. Because ROS act as a double-edged sword, cells employ redox sensor proteins to precisely recognize the intracellular ROS levels and maintain cellular redox homeostasis [6,7].

The cysteine (Cys) residues of redox sensitive proteins effectively perceive the redox change, given their nucleophilic properties, and transduce this change as a signal to downstream molecules, thus activating downstream signaling processes to control protein activity, gene transcription, and hormone signaling pathways [8,9]. Thus, the Cys residues act as a redox switch.

Although the redox status of proteins appears to be a central element in the control of many physiological processes, only a limited number of sensors have been identified to date [10]. Therefore, scientists have been searching for new redox sensor proteins by computational and bioinformatics approaches [11,12]. However, the theoretical approach used to discover catalytic redox sensors is highly uncertain, which highlights the importance of combining the theoretical and experimental procedures to identify novel redox sensors [10]. A clue for the identification of redox sensor candidates is derived from the sporadic revelation of selenocysteine (Sec)–Cys pairs in sensor protein families [11]. One such family consists of thioredoxin (TRX) and TRX reductase proteins, whose active site Cys residues probably originated from Sec in their primitive forms [13]. Thus, Cys and Sec are sporadically detected at the active site of TRXs in various organisms [14]. Regulated reversal of cysteine oxidation is dependent on activities of the conserved superfamily of TRXs that function as cysteine reductases. The plant immune system recruits specific TRXs that have the potential to functionally regulate numerous immune signaling proteins [15]. Additionally, TRXs participate in diverse signaling pathways through the regulation of protein S-nitrosation in plants [16]. Another group of redox proteins comprises nucleoredoxins (NRXs), which contain three tandemly arranged TRX-like modules, and localize to both the nucleus and cytoplasm [17]. Notably, the *Arabidopsis thaliana* genome encodes two NRX isotypes, AtNRX1 and AtNRX2 [17,18]. While the function of AtNRX2 remains poorly characterized, the antioxidant property of AtNRX1 was recently reported [19]. AtNRX1 protects plants from oxidative stress by forming mixed disulfide bonds with antioxidant enzymes and by supplying reducing power to its targets.

In the present study, we selected NRXs as candidate redox sensor proteins and investigated their physiological significance in *Arabidopsis*. We demonstrate that AtNRX1 exhibits redox-dependent structural modifications, which activate defense responses against the fungal pathogen, *Alternaria brassicicola*, via jasmonic acid (JA) signaling. This novel JA signaling cascade links the redox status of NRXs with phytohormone signaling and stress tolerance in plants. Only the results obtained with AtNRX1 have been reported in this study; the results of AtNRX2 will be reported in a subsequent manuscript because AtNRX2 shares less than 30% amino acid sequence identity with AtNRX1 and exhibits substantially different properties than AtNRX1.

## 2. Results

### 2.1. AtNRX1 Exhibits Redox-Dependent Structural Modification

**Figure 1:**
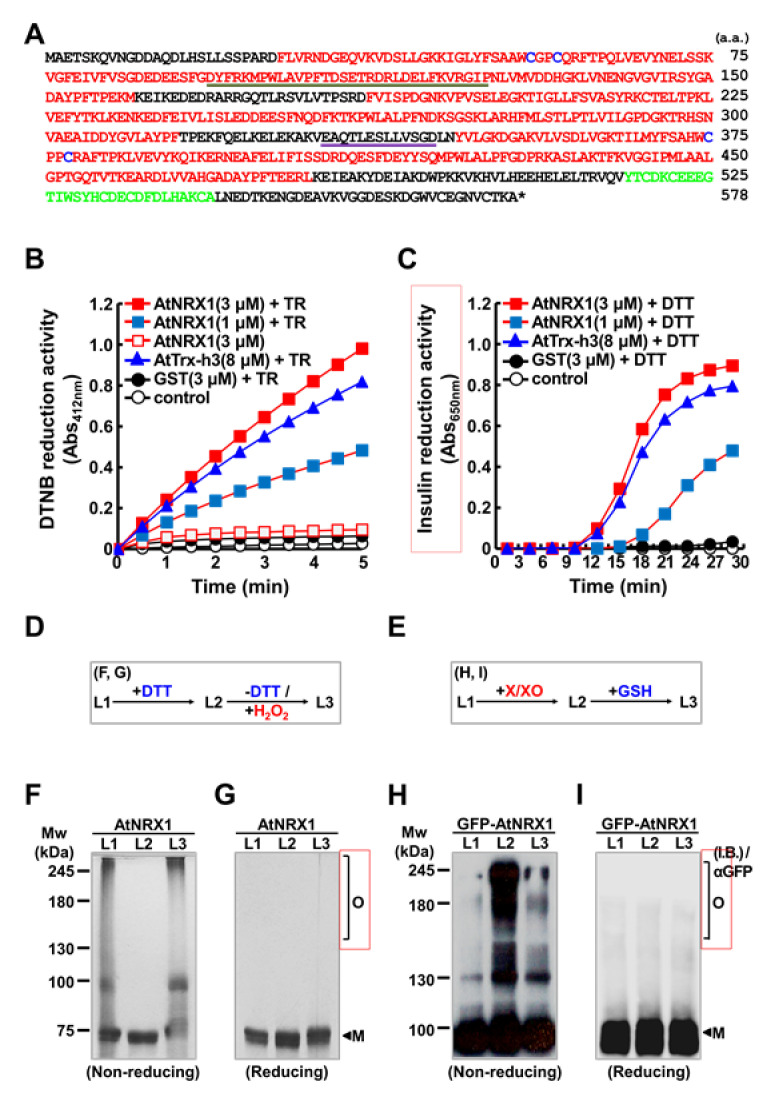
Structural and functional characterization of AtNRX1. (**A**) Amino acid sequence of AtNRX1. Thioredoxin (TRX) domains are indicated in red, and the divergent C1 domain is indicated in green. Four conserved cysteine (Cys) residues in TRXa and TRXa′ domains are denoted in blue. The bipartite nuclear localization signal (NLS; underlined in brown) and nuclear export signal (NES; underlined in pink) were predicted by NLS mapper (http://nls-mapper.iab.keio.ac.jp/cgi-bin/NLS_Mapper_form.cgi) and Wregex (http://wregex.ehubio.es), respectively. (**B**,**C**) Disulfide reductase activity of AtNRX1 tested using 5 mM DTNB (**B**) and insulin (**C**) as substrates. In (**B**), the reductase activity of AtNRX1 was assessed by measuring the absorbance at 412 nm (Abs_412_) in the presence of NADPH and thioredoxin reductase (TR) along with 1 μM AtNRX1 (■), 3 μM AtNRX1 (■), 3 μM GST (●), or 8.35 μM AtTRX-h3 (▲); 3 μM AtNRX1 in the absence of TR (□) and substrate alone (control) in the presence of TR (○) were included for comparison. In (**C**), the reductase activity of AtNRX1 was measured in the presence of 0.5 mM DTT and 1 μM AtNRX1 (■), 3 μM AtNRX1 (■), 3 μM GST (●), or 8.35 μM AtTRX-h3 (▲); insulin reduction in the presence of only 0.5 mM DTT (○) was used as a control. (**D**–**I**) Redox-dependent reversible structural modification of AtNRX1 (from monomer to oligomer). (**D**,**E**) Schemes showing the sequential treatment of the recombinant AtNRX1 protein in vitro (**D**) and GFP-AtNRX1 (**E**) in tobacco leaves with reducing agents (DTT or GSH), oxidizing agents (H_2_O_2_), and xanthine/xanthine oxidase (X/XO) (**E**). (**F**–**I**) Redox-dependent structural switch of AtNRX1 in vitro (**F**,**G**) and GFP-AtNRX1 isolated from tobacco leaves (**H**,**I**). Proteins were loaded in three lanes (L1–L3) on non-reducing (**F**,**H**) and reducing (**G**,**I**) acrylamide gels and separated by SDS-PAGE. The gels were subjected to silver staining (**F**,**G**) and immunoblotting with anti-GFP antibody (**H**,**I**). Numbers on the left of (**F**) and (**H**) represent the molecular weight (MW) of the protein marker. Monomer (M) and oligomers (O) of AtNRX1 are indicated on the right side of panels (**G**) and (**I**).

Given that AtNRX1 is a redox protein containing multiple TRX domains (Figure 1A), we first measured the reductase activity of recombinant AtNRX1 protein expressed in *Escherichia coli* using the universal substrates, including 5,5-dithio-bis-(2-nitrobenzoic acid) (DTNB) and insulin. AtNRX1 worked with thioredoxin reductase (TR) to reduce DTNB, as expected. Additionally, in the presence of 1,4-dithiothreitol (DTT), AtNRX1 reduced insulin in a concentration-dependent manner, thus confirming its reductase activity (Figure 1A,B). In assays carried out with both DTNB and insulin, the reductase activity of AtNRX1 was at least 2.7-fold higher than that of AtTrx-h3. Redox sensor proteins, such as 2-Cys peroxiredoxin, glutathione peroxidase (GPx), and non-expressor of PR1 (NPR1), switch their quaternary structures in response to external stimuli through the reversible exchange of dithiol and disulfide bonds [9,20,21]. Considering the structural properties of AtNRX1, a TRX family protein (Figure 1A), we hypothesized that AtNRX1 undergoes redox-dependent structural modifications. To test this hypothesis, we analyzed the oligomerization of AtNRX1 after sequential treatment with reducing and oxidizing agents (Figure 1D–I). The recombinant AtNRX1 protein purified in vitro and the green fluorescence protein (GFP)-AtNRX1 fusion protein transiently expressed in tobacco (*Nicotiana benthamiana*) leaves via agroinfiltration were treated according to the schemes shown in Figure 1D,E, respectively. Subsequently, the status of recombinant AtNRX1 and GFP-AtNRX1 proteins was examined on non-reducing (Figure 1F,H) and reducing (Figure 1G,I) acrylamide gels by sodium dodecyl sulfate-polyacrylamide gel electrophoresis (SDS-PAGE). The results showed that untreated AtNRX1 consisted of multiple monomeric forms and discrete oligomeric forms (Figure 1F). However, DTT treatment dissociated the AtNRX1 oligomers into monomers (Figure 1F). Upon the removal of DTT with a PD-10 desalting column and subsequent treatment with hydrogen peroxide (H_2_O_2_), the oligomeric structure of AtNRX1 was restored (Figure 1F). Analysis of untreated, DTT-treated, and H_2_O_2_-treated samples by reducing SDS-PAGE indicated that the level of the recombinant AtNRX1 protein was not changed by the redox treatments (Figure 1G). These data suggest that the quaternary structure of AtNRX1 reversibly switches between monomeric and oligomeric forms in a redox-dependent manner. The redox-dependent structural changes of the recombinant AtNRX1 observed in vitro were confirmed in planta (Figure 1F,G). GFP-AtNRX1 transiently expressed in tobacco leaves via agroinfiltration reversibly switched its structure upon treatment with xanthine/xanthine oxidase (X/XO) and subsequent treatment with GSH (Figure 1H,I). The protein extracts of plants in the mock treatment primarily consisted of GFP-AtNRX1 monomers and a small amount of the corresponding oligomers (Figure 1H). However, the amount of GFP-AtNRX1 oligomers increased significantly in tobacco leaves treated with X/XO for 3 h (Figure 1H) compared with the mock sample. Furthermore, the highly abundant GFP-AtNRX1 oligomers, produced upon treatment with X/XO, mostly dissociated into monomers by subsequent treatment with GSH (Figure 1H). These results indicate that the structural modification of AtNRX1 in planta is reversibly regulated by X/XO and intracellular redox status.

### 2.2. Cys Residues in the TRX Domains Are Essential for the Oligomerization of AtNRX1

**Figure 2:**
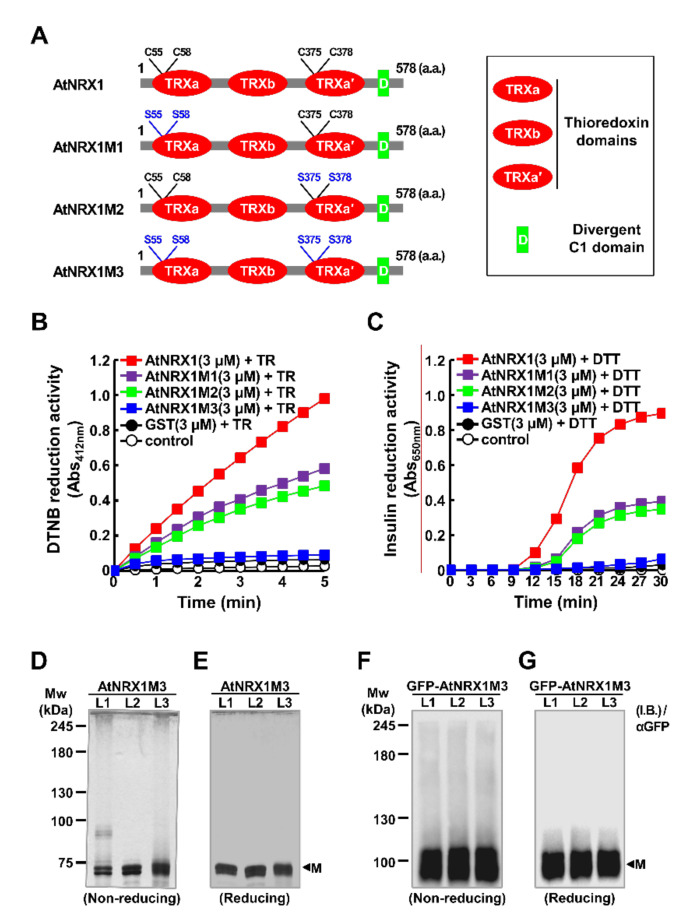
Effect of active site Cys residues on the structural and functional properties of AtNRX1. (**A**) Structural depictions of the wild-type (WT) and mutant AtNRX1s. Three TRX domains (TRXa, TRXb, and TRXa′) and one DC1 domain are depicted. The active site Cys residues and the substituted serine (Ser) residues of AtNRX1s are indicated in black and blue, respectively. (**B**,**C**) Disulfide reduction activity of the WT and mutant AtNRX1s using 5 mM DTNB (**B**) and insulin (**C**) as substrates. In (**B**), the reduction activity of AtNRX1 proteins was measured by the presence of NADPH, TR, and 3 μM AtNRX1 (■); 3 μM AtNRX1M1 (■); 3 μM AtNRX1M2 (■); 3 μM AtNRX1M3 (■); or 3 μM GST (●). Substrate alone in the presence of TR (○) was used as a control. In (**C**), the reductase activity of AtNRX1 was measured in the presence of 0.5 mM DTT and 3 μM AtNRX1 (■), 3 μM AtNRX1M1 (■), 3 μM AtNRX1M2 (■), 3 μM AtNRX1M3 (■), or 3 μM GST (●); insulin reduction in the presence of only 0.5 mM DTT (○) was used as a control. (**D**–**G**) Redox-dependent structural properties of AtNRX1M3 in vitro (**D**,**E**) and GFP-AtNRX1M3 isolated from tobacco leaves (**F**,**G**). Proteins were loaded in three lanes (L1–L3) on non-reducing (**D**,**F**) and reducing (**E**,**G**) acrylamide gels and separated by SDS-PAGE.

AtNRX1 possesses three TRX domains (Figure 2A), of which two are active (a and a′), and one is inactive (b). According to previous reports, TRX domains often mediate the oligomerization of proteins containing them, and Cys residues in the active TRX domains are required for this process [22,23]. Therefore, we decided to investigate the biological relevance of Cys residues in the TRX domains of AtNRX1 (Figure 2A). To this end, we produced three mutant variants of AtNRX1: AtNRX1M1, in which two active site Cys residues at amino acid positions 55 and 58 (C55 and C58, respectively) of the TRXa domain were replaced by Ser residues; AtNRX1M2, in which C375 and C378 in the active site of TRXa′ domain were replaced by Ser residues; and AtNRX1M3, harboring all four Cys-to-Ser substitutions (C55S), (C58S), C375S, and C378S). Recombinant wild-type (WT) and mutant AtNRX1 proteins were produced in *E. coli*, and the reductase activity of each of these proteins was measured using the universal substrates, DTNB and insulin (Figure 2B,C). The WT AtNRX1 worked with TR and DTT to reduce DTNB and insulin, respectively, thus confirming its reductase activity. Among the mutant proteins, both AtNRX1M1 and AtNRX1M2 reduced DTNB and insulin, although the reductase activity of these two mutant proteins was only 50% of that of AtNRX1. On the other hand, AtNRX1M3 showed no reductase activity. Next, we investigated the role of active site Cys residues in the oligomerization of AtNRX1 using AtNRX1M3. Purified recombinant AtNRX1M3 and transiently generated GFP-AtNRX1M3 in tobacco leaves were treated according to the schemes shown in Figure 1D,E, respectively, and the redox status of these proteins was then examined on non-reducing (Figure 2D,H) and reducing (Figure 2E,I) acrylamide gels by SDS-PAGE. The results showed that both recombinant AtNRX1M3 in vitro and GFP-AtNRX1M3 in vivo (Figure 2D,H) existed as monomers, regardless of their redox status, unlike recombinant AtNRX1 (Figure 1F,H), indicating that the active site Cys residues of AtNRX1 play a critical role in its redox-dependent structural switching.

### 2.3. Subcellular Localization of AtNRX1 and AtNRX1M3

**Figure 3:**
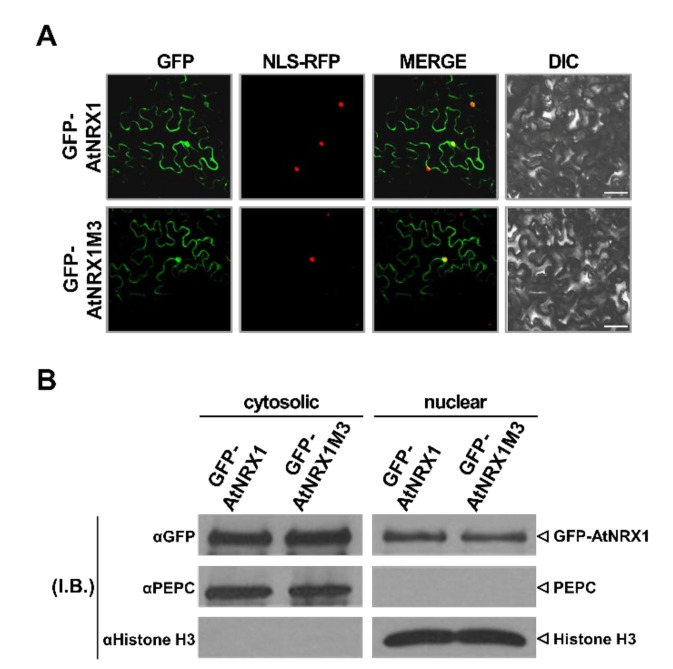
Subcellular localization analysis of AtNRX1 and AtNRX1M3. (**A**) Subcellular localization patterns of GFP-AtNRX1 and GFP-AtNRX1M3 in agroinfiltrated tobacco leaves. Tobacco leaves were co-infiltrated with *NLS-RFP* and *GFP-AtNRX1* or *GFP-AtNRX1M3*. The fluorescence signals of GFP (left panel) and RFP (second panel) were observed by confocal microscopy of epidermal cells at 3 days post-infiltration (dpi). To confirm the nuclear localization of GFP-AtNRX1 and GST-AtNRX1M3, GFP and RFP images were merged (third panel). Differential interference contrast (DIC) images (right panel) of the fields are shown. Scale bars = 100 μm. (**B**) Fractionation analysis of GFP-AtNRX1 and GFP-AtNRX1M3 produced in agroinfiltrated tobacco leaves. The *GFP-AtNRX1* and *GFP-AtNRX1M3* constructs were infiltrated into tobacco leaves. Total proteins were extracted from leaves, and the cytosolic and nuclear fractions were separated. The protein level of GFP-AtNRX1 was determined by immunoblotting (I.B.) with anti-GFP antibody. The degree of fraction enrichment was determined using antibodies specific to marker proteins (anti-histone H3, nuclear marker; anti-PEPC, cytosolic marker). Experiments were repeated three times with similar results.

Bioinformatics analysis using the NLS mapper (http://nls-mapper.iab.keio.ac.jp/cgi-bin/NLS_Mapper_form.cgi) revealed a putative bipartite type nuclear localization signal (NLS) at the N-terminal end of AtNRX1, while analysis with Wregex (http://wregex.ehubio.es) indicated a nuclear export signal (NES) in the middle of the sequence (Figure 1A). The dual localization of AtNRX1 to the cytosol and nucleus, reported previously [17], was confirmed in our study (Supplementary Materials Figure S1). Because AtNRX1M3 showed different characteristics than AtNRX1 in terms of its reductase function (Figure 1) and stress sensitivity (Figure 2), we compared its subcellular localization pattern with that of AtNRX1. Tobacco leaves were co-infiltrated with *NLS-RFP* and *GFP-AtNRX1* or *GFP-AtNRX1M3* constructs. After 3 days, the fluorescence signals of GFP and RFP were visualized in epidemic cells under a confocal microscope (Figure 3A). Both GFP-AtNRX1 and GFP-AtNRX1M3 co-localized to the nucleus along with NLS-RFP. Additionally, in our fractionation experiments, both GFP-AtNRX1 and GFP-AtNRX1M3 were detected in cytoplasmic as well as nuclear fractions of agroinfiltrated tobacco leaves (Figure 3B). These results indicate that both AtNRX1 and AtNRX1M3 show identical subcellular localization patterns.

### 2.4. Expression Analysis of AtNRX1

**Figure 4:**
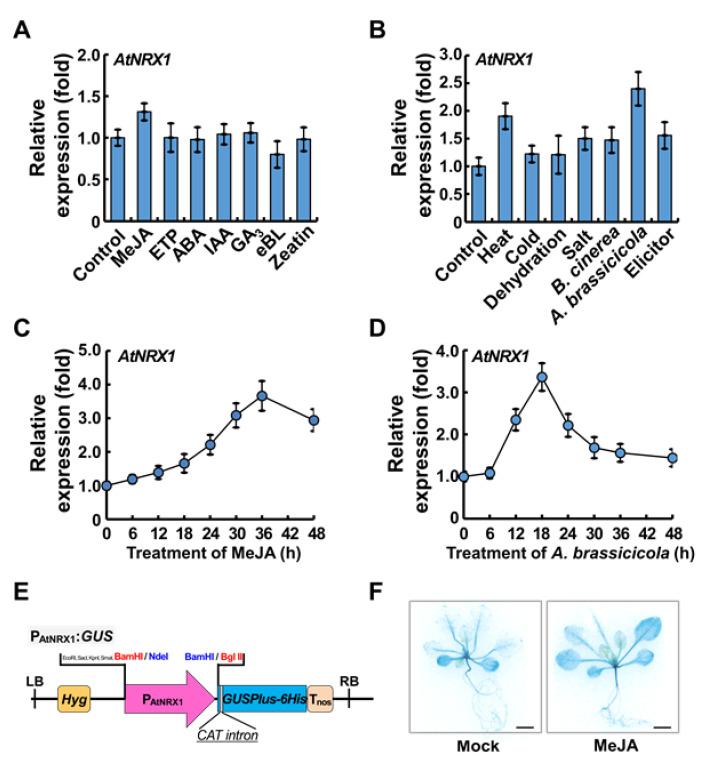
Effect of various stresses and plant hormones on *AtNRX1* expression. (**A**) Effect of plant hormones on *AtNRX1* expression. Seedlings were treated with 10 µM methyl jasmonate (MeJA), 10 µM ethephon (ETP), 10 µM abscisic acid (ABA), 1 µM indole-3-acetic acid (IAA), 1 µM gibberellin A_3_ (GA_3_), 10 nM 24-epibrassinolide (eBL), and 20 µM t-zeatin (zeatin) for 3 h. (**B**) *AtNRX1* expression in response to various stresses. Seedlings were treated with high temperature (37 °C for 2 h), low temperature (4 °C for 6 h), dehydration (air-dried for 12 h), salt (300 mM NaCl), fungal pathogens (*Botrytis cinerea* and *Alternaria brassicicola*), and a peptide elicitor. (**C**) *AtNRX1* expression in response to JA. Seedlings were treated with MeJA, and samples were collected at the indicated time-points. (**D**) *AtNRX1* expression in response to *A. brassicicola* infection. Seedlings were infected with *A. brassicicola*, and samples were collected at the indicated time-points. In (**A**–**D**), total RNA was isolated from stress- or hormone-treated 2-week-old whole seedlings, and *AtNRX1* expression was determined by quantitative real-time PCR (qRT-PCR) using *ACTIN2* (*ACT2*), *Ubiquitin1* (*UBQ1*), and *UBQ10* as endogenous references. Data represent mean ± standard error (SE; *n* = 3). Error bars indicate standard error of the mean. (**E**) A schematic diagram of the *P_AtNRX1_*:*GUS* construct. RB, right border; LB, left border; P35S, cauliflower mosaic virus (CaMV) *35S* promoter; P_AtNRX1_, *AtNRX1* gene promoter; Hyg, hygromycin phosphotransferase coding region; *GUSPlus-6×His*, β-glucuronidase gene fused to six histidine codons; *CAT intron*, catalase gene intron; T_nos_, nopaline synthase terminator. The insertion position of the P_AtNRX1_ in the vector is indicated by the restriction enzyme sites, *Bam*HI/*Nde*I and *Bam*HI/*Bgl*II. (**F**) Histochemical analysis of *P_AtNRX1_*:*GUS* lines after mock and MeJA treatments. Scale bar = 3 mm.

To identify a specific plant hormone that induces the expression of *AtNRX1* (Figure 4A), we analyzed its expression in *Arabidopsis* plants treated with a wide variety of plant hormones including 10 µM methyl jasmonate (MeJA), 10 µM ethephon (ETP), 10 µM abscisic acid (ABA), 1 µM indole-3-acetic acid (IAA), 1 µM gibberellin A_3_ (GA_3_), 10 nM 24-epibrassinolide (eBL), and 20 µM t-zeatin (zeatin). The transcript level of *AtNRX1* showed the greatest increase in the MeJA treatment; however, no significant change was detected with other hormones. Additionally, to determine whether AtNRX1 is associated with abiotic/biotic stress response, we analyzed the expression of *AtNRX1* in 14-day-old WT *Arabidopsis* Columbia (Col-0) plants treated with heat stress (37 °C for 2 h), cold stress (4 °C for 6 h), dehydration (air-dried for 12 h), salt stress (300 mM NaCl), fungal pathogens (*Botrytis cinerea* and *A. brassicicola*), and a peptide elicitor (Figure 4B). The transcript level of *AtNRX1* was markedly elevated by heat stress and *A. brassicicola* infection, and mildly increased by cold, dehydration, salt, peptide elicitor, and *B. cinerea*. Because *AtNRX1* expression was greatly increased by necrotic fungal pathogens and MeJA, we also monitored its expression by time pulses after MeJA treatment and *A. brassicicola* infection (Figure 4C,D). The relative transcript level of *AtNRX1* gradually increased after treatment with both *A. brassicicola* and MeJA, reaching a peak at 36 h and 18 h post-treatment, respectively. Similarly, our histochemical analysis of transgenic *Arabidopsis* plants expressing the *ß-glucuronidase* (*GUS*) gene under the control of the *AtNRX1* promoter (*P_AtNRX1_*:*GUS*) showed MeJA-induced expression of *AtNRX1* (Figure 4E,F).

### 2.5. AtNRX1 Negatively Controls the Defense Response against Necrotic Fungal Pathogens

**Figure 5:**
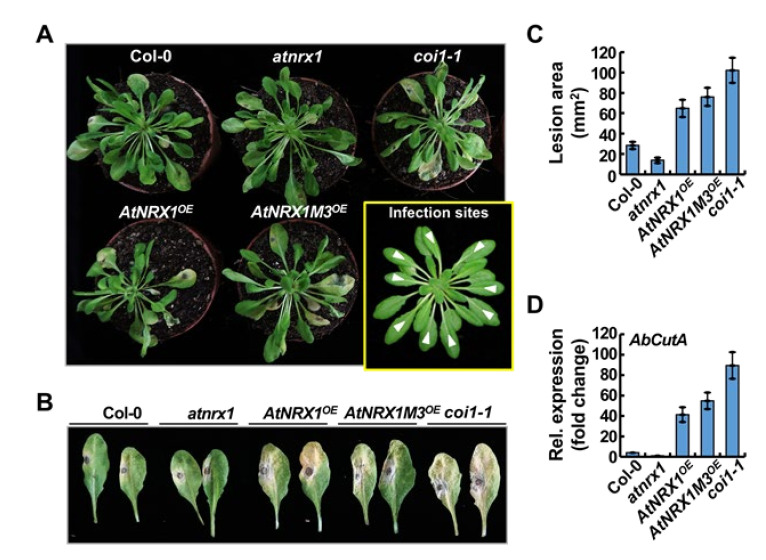
AtNRX1 negatively regulates resistance to *A. brassicicola* in *Arabidopsis*. (**A**) Susceptibility of *Arabidopsis* plants to *A. brassicicola* infection at 7 dpi. Leaves were drop-inoculated with 5 µL (5 × 10^5^ spores/mL) of the fungal spore suspension. (**B**) Photographs showing lesions on leaves at 4 dpi. (**C**) Average size of at least 60 lesions on the leaves of 30 plants, with at least three replicates. Data represent mean ± SE (*n* = 3). (**D**) Relative expression level of *A. brassicicola Cutinase A* (*AbCutA*) in inoculated plants analyzed by qRT-PCR at 4 dpi.

JA signaling regulates various aspects of plant physiology including plant growth, development, and stress responses. Defense signaling against fungal pathogens or wounding stress is mediated by various combinations of JA-responsive transcription factors (TFs) and their activity regulators, such as JASMONATE ZIM-DOMAIN (JAZ) proteins [24]. Specific interaction between JA signaling TFs and other downstream transcriptional regulators fine-tunes the output of JA signaling during particular plant physiological responses [25]. Based on the results indicating that the expression of *AtNRX1* is regulated by JA and fungal pathogens (described above), we investigated the physiological roles of AtNRX1 and the underlying molecular mechanisms using the T-DNA insertion mutant, *atnrx1* (SALK_113401) (Figure S2A), and transgenic *Arabidopsis* lines overexpressing *AtNRX1* or *AtNRX1M3* under the control of the cauliflower mosaic virus *35S* (*P35S*) promoter in the WT (Col-0) background (Figure S2B). The results of semi-quantitative reverse transcription PCR (sqRT-PCR) revealed that the expression of *AtNRX1* and *AtNRX1M3* was much higher in *AtNRX1* overexpression (*AtNRX1^OE^*) and *AtNRX1M3* overexpression (*AtNRX1M3^OE^*) lines, respectively, than in WT plants, and no signal was detected in the *atnrx1* mutant (Figure S2B). By contrast, transcript levels of *Actin2* (*ACT2*; At3g18780), used as a control, were nearly identical in all samples (Figure S2C). Additionally, the growth phenotypes of 3-week-old WT, *atnrx1*, *AtNRX1^OE^*, and *AtNRX1M3^OE^* plants were also similar (Figure S2D). Next, we analyzed disease symptoms and fungal growth in 6-week-old *Arabidopsis* plants infected with *A. brassicicola*. Infected leaves of *AtNRX1^OE^* and *AtNRX1M3^OE^* plants showed significantly more severe disease symptoms (Figure 5A) and larger necrotic lesions (Figure 5B,C) than those of WT plants, indicating that overexpression of *AtNRX1* and *AtNRX1M3* increased the susceptibility of Arabidopsis to *A. brassicicola* infection. By contrast, *atnrx1* plants showed stronger resistance to the necrotrophic fungal pathogen than WT plants (Figure 5A–C). To quantify the proliferation of *A. brassicicola* in infected Arabidopsis plants, the relative expression level of the *A. brassicicola Cutinase A* (*AbCutA*) gene was measured by quantitative real-time PCR (qRT-PCR) and normalized relative to the expression levels of *Arabidopsis ACT2*, *Ubiquitin 1* (*UBQ1*), and *UBQ10* genes (used as endogenous references) (Figure 5D). In the *atnrx1* mutant, the expression level of *AbCutA* remained at a low level, similar to that in WT plants. However, consistent with the increased disease symptoms, *AtNRX1^OE^* and *AtNRX1M3^OE^* plants showed markedly rapid proliferation of *A. brassicicola* than WT plants. Thus, overexpression of *AtNRX1* and *AtNRX1M3* increased the susceptibility of *Arabidopsis* to *A. brassicicola* infection. Furthermore, based on the *AbCutA* mRNA level, *AtNRX1M3^OE^* plants appeared to be more susceptible to *A. brassicicola* infection than *AtNRX1^OE^* plants (Figure 5D).

### 2.6. AtNRX1 Negatively Regulates the Expression of PDF1.2

**Figure 6:**
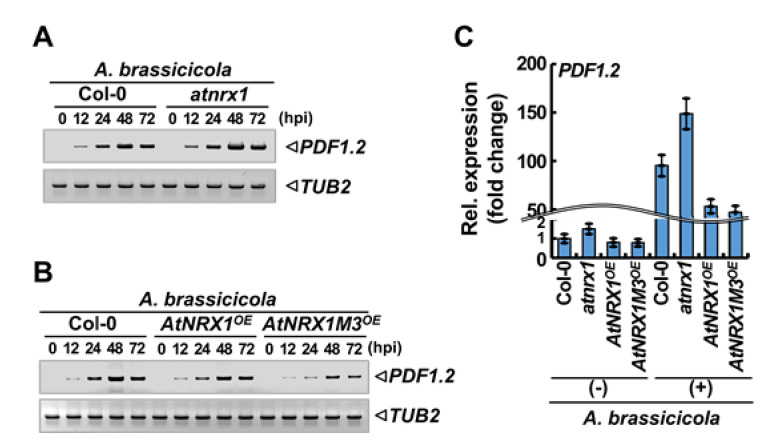
AtNRX1 negatively regulates JA-mediated *PDF1.2* expression in *Arabidopsis*. (**A**,**B**) Semi-quantitative reverse transcription PCR (sqRT-PCR) analysis of *PDF1.2* expression in Col-0 and *atnrx1* (**A**) or Col-0, *AtNRX1^OE^*, and *AtNRX1M3^OE^* (**B**) plants infected with *A. brassicicola*. Mature leaves of 4-week-old *Arabidopsis* plants were inoculated with 3 μL droplets of *A. brassicicola* spore suspension (10^6^ spores/mL). Total RNA was isolated at the indicated time-points, and expression of *PDF1.2* and *TUB2* (control) was analyzed by sqRT-PCR. (**C**) Relative expression of *PDF1.2* in Col-0, *atnrx1*, *AtNRX1^OE^*, and *AtNRX1M3^OE^* plants before (−) or after (+) *A. brassicicola* infection. Droplets (3 μL) of the spore suspension (10^6^ spores/mL) were applied to mature leaves. Total RNA was isolated from the leaves, and expression of *PDF1.2* relative to that of *ACT2*, *UBQ1*, and *UBQ10* was determined by qRT-PCR at 48 h post-inoculation (hpi). Data represent mean ± SE (*n* = 3).

Compared with WT plants, *atnrx1* mutant plants were more resistant to *A. brassicicola* infection, whereas *AtNRX1^OE^* plants were more susceptible. This indicates that AtNRX1 negatively regulates the plant response to the necrotrophic fungal pathogen. To investigate how AtNRX1 controls the response to *A. brassicicola* infection and JA signaling [26], we examined the transcript level of *PLANT DEFENSIN 1.2* (*PDF1.2*), a well-characterized JA signaling gene involved in defense against necrotrophic fungal pathogens, in WT, *atnrx1*, *AtNRX1^OE^*, and *AtNRX1M3^OE^* plants by time pulses after *A. brassicicola* infection by sqRT-PCR using sequence-specific primers. Accumulation of *PDF1.2* transcripts increased by *A. brassicicola* infection in WT plants, with the highest accumulation at 2 days post-infection (dpi) (Figure 6A,B). Compared with WT plants, the expression of *PDF1.2* was enhanced in *atnrx1* mutant plants (Figure 6A) but reduced in *AtNRX1^OE^* and *AtNRX1M3^OE^* plants (Figure 6B). We also analyzed the expression of *PDF1.2* in WT, *atnrx1*, *AtNRX1^OE^*, and *AtNRX1M3^OE^* by qRT-PCR before and after *A. brassicicola* infection. The results showed that *PDF1.2* expression was significantly induced by *A. brassicicola* infection in WT plants at 2 dpi. Compared with the WT, the expression of *PDF1.2* was approximately 56% higher in *atnrx1* plants, whereas 44% and 50% lower in *AtNRX1^OE^* and *AtNRX1M3^OE^* plants, respectively (Figure 6C).

## 3. Discussion

To coordinate environmental cues with intracellular responses in plants, redox sensors containing catalytic Cys-thiols sensitively perceive the alteration in ROS levels and transduce this information to the corresponding target systems through post-translational modifications [27,28,29]. Thus, ROS-mediated shifts in the cellular redox potential are thought to constitute a central mechanism that regulates systemic networks of plant metabolism, phytohormone signaling, and plant physiology [2,3,30]. For example, the drought resistance of plants is controlled by the interplay between redox changes and phytohormone signaling through the ethylene and ABA networks [31,32]. The defense response and stomatal closure of plants are also controlled by the crosstalk between the redox status and plant hormones such as salicylic acid (SA), JA, and ABA [33]. Root architecture is tightly regulated by the orchestration of redox signals and numerous plant hormones [13,34]. JA-induced changes in gene expression are regulated by the abundance of ascorbate and GSH pools [35,36]. Although these and other examples indicate that changes in ROS levels regulate the function of phytohormone (SA, ethylene, JA) signaling pathways and their metabolic and physiological outputs [2,37], the molecular mechanisms that determine how the redox changes regulate these processes remain poorly understood.

JA and its derivatives, including MeJA, jasmonoyl isoleucine (JA-Ile), cis-12-oxophytodienoic acid (OPDA), and cis-jasmone, are produced by both healthy and wounded plants [38,39,40]. In addition, these hormones accumulate to high levels in response to a wide variety of external stimuli, such as herbivorous insects, necrotrophic pathogens, and other biotic/abiotic stresses [41,42]. JA and its derivatives, most notably JA-Ile, regulate a wide spectrum of plant physiological processes, such as root growth inhibition, plant development, pathogen resistance, male sterility, trichome initiation, and anthocyanin accumulation, by regulating diverse sets of intracellular signaling pathways and reprogramming downstream target genes [42,43]. The molecular basis of JA signaling was initially uncovered through the characterization of the SCF^COI1^ protein [44]. The JA insensitive *coi1-1* mutant shows multiple phenotypes. Extensive forward genetic screening, coupled with knockout or gain-of-function mutation experiments, identified several positive and negative regulators of JA signaling [45]. Upon binding to bioactive JAs, the JA receptor, SCF^COI1^, binds to and ubiquitinates negative regulators of JA signaling, namely, JAZ proteins. The JAZ proteins are subsequently degraded by the 26S proteasome, which allows JA-responsive TFs to regulate the expression of JA-responsive genes [44,46].

Despite the exclusive JA signaling pathway mediated by JAZ-TFs (e.g., MYC/MYB TFs) identified from plants, it has also been discovered that there are several mutants showing partially independent phenotypes associated with JAZ-TFs-mediated JA signaling, as well as several mutant phenotypes associated with JAZ-TFs that are weaker than those of *coi1-1* [47,48]. The results suggest that JAZ-TFs are not the only component of the JA signaling pathway; instead, other TFs or regulatory proteins also regulate the diverse JA signaling outputs. To account for this possibility, several hypotheses have been proposed, including the heterodimerization of JA-responsive TFs with other types of TFs, and the recruitment of other co-regulators that compete to bind to the TFs. To gain a more complete understanding of how JA signaling regulates its diverse metabolic and physiological outputs, it is important to identify novel JA signaling regulators or TFs [26,49].

**Figure 7:**
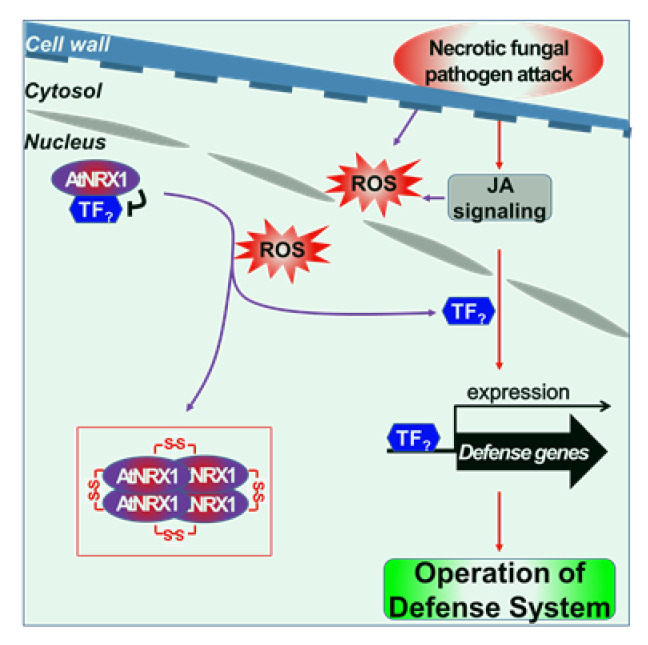
Model depicting the mode of action of AtNRX1 in the JA-dependent defense system under normal (no stress) and biotic stress conditions. In this model, AtNRX1 inhibits the JA-dependent expression of defense-related genes under normal conditions. However, the external attack by necrotrophic fungal pathogen simultaneously produces JA and ROS. The associated accumulation of intracellular ROS results in the oxidation of the active site Cys residues of AtNRX1, which behave as a redox sensor and induce the structural modification (oligomerization) of AtNRX1 monomers. The decreased affinity of oligomeric AtNRX1 for the TF_?_ (an unknown factor in the nucleus, such as a TF mediating JA signaling) results in the de-repression of TF_?_, thus activating JA signaling and altering the expression profile of a subset of JA-responsive genes.

In this study, we identified a novel regulator of a major JA signaling component, AtNRX1, and summarize our results in a working model (Figure 7). Under normal conditions (no stress), AtNRX1 exists as a reduced monomer, which inhibits the expression of defensive-related genes via JA signaling. In this process, AtNRX1 does not act as a TF; therefore, it is assumed that AtNRX1 works together with an unknown factor in the nucleus, such as a TF mediating JA signaling (TF_?_ in Figure 7). The nuclear localization of AtNRX1, reported previously [17], was confirmed in the current study (Figure S1). A variety of external stimuli simultaneously produce JA and ROS [50]. The associated accumulation of intracellular ROS results in the oxidation of the active site Cys residues of AtNRX1, which behave as a redox sensor and induce the structural modification (oligomerization) of AtNRX1 monomers. The decreased affinity of oligomeric AtNRX1 for the TF_?_ results in the de-repression of TF_?_, thus activating JA signaling and altering the expression profile of a subset of JA-responsive genes [41]. If the plant defense system under attack by necrotrophic fungal pathogens is compared with the mechanical system of a rifle, AtNRX1 would be considered a safety pin. In other words, if the redox-dependent oligomerization of AtNRX1 causes its dissociation from TF_?_, the JA signal triggers the TF_?_ to activate the expression of defensive-related genes such as *PDF1.2* that attack pathogens. This model shares some similarity with the redox-dependent regulation of Wnt/β-Catenin signaling by the cytosolic form of NRX in mammalian cells [51]. Wnt signaling-mediated changes in intracellular redox status cause the oxidation of NRX and its dissociation from Disheveled 1 (Dvl1), a central negative regulator of Wnt signaling. However, in contrast to the nuclear AtNRX1 protein, cytosolic mammalian NRX does not show redox-dependent structural switching under oxidized conditions. According to a recent study of human diseases, Thioredoxin interacting protein (TXNIP) has been identified as an important physiological inhibitor of the TRX redox system playing an important role in redox regulation in cells [52]. Likewise, exploring the various partner proteins of AtNRX1 and studying their functions will also greatly help to better understand the intracellular role of AtNRX1. Given that the structural shift of redox proteins, including redox-mediated monomer-to-oligomer transition, can confer specific functions to proteins in their discrete structural states [53,54], it will be of interest to determine whether the oligomerized AtNRX1 performs these functions independent of the TF_?_ in plant nuclei. TF_?_, which is inhibited by AtNRX1 and is expected to promote the expression of defense-related genes such as *PDF1.2* by mediating JA signaling, should be identified in future research. Our findings are somewhat reminiscent of redox-dependent regulation of SA signaling, in which the transcription cofactor, NPR1, induces ROS-mediated structural modification of oligomers into monomers, and the nuclear form of NPR1 binds to TGACG-motif binding (TGA) TFs and triggers transcriptional reprogramming [21,55,56].

In the SDS PAGE analysis, AtNRX1 proteins showed various protein band patterns in addition to oligomer formation. Two close bands appeared for monomeric AtNRX1 in Figure 1F,G and for monomeric AtNRX1M3 in Figure 2D,E. Additionally, a protein signal (approx. at 90 kDa) is observed in Figure 2D under non reducing (L1) conditions. These phenomena are expected to be caused by protein modification by various post-translational modifications such as nytrosylation, phosphorylation, acetylation, metal-binding, etc. We hope that the post-translational modifications of AtNRX1 by other ways in addition to oxidation/reduction of catalytic Cys-thiols and their functions will be studied and revealed in more detail.

Increasing evidence shows that ROS-mediated redox signaling is interwoven with phytohormone [1]. Consistent with this finding, we here demonstrated that AtNRX1 acts as a redox sensor that coordinates a subset of JA signaling proteins in plants. In particular, our results indicate a role for the AtNRX1/–TF_?_ module in regulating a subset of the JA response that is critical for effective biotic defense against a necrotrophic fungal pathogen. However, our results suggest that this module is relatively less important for the developmental and growth responses of plants. In our study, according to the expression analysis results of *PDF1.2*, the typical disease resistance gene against necrotic fungal pathogens, the disease resistance of *atnrx1* mutant is due to improved activation of JA signaling, while the disease susceptibility of *AtNRX1^OE^* and *AtNRX1M3^OE^* plants appears to be due to delayed activation of JA signaling. In other words, *AtNRX1^OE^* and *AtNRX1M3^OE^* plants are disease-sensitive because excessively accumulated AtNRX1 or AtNRX1M3 proteins continue to impede activation of JA signaling even in the event of pathogen infection, delaying the expression of defense genes such as PDF1.2. If we compare *AtNRX1^OE^* and *AtNRX1M3^OE^*, the expression of *PDF1.2* in *AtNRX1M3^OE^* is relatively less than that of *AtNRX1^OE^* (Figure 6A,B). This is because the AtNRX1M3 will not undergo redox-dependent structural changes, thus more severely preventing the activation of JA signaling than AtNRX1. Nonetheless, closer examination of the interaction between *Arabidopsis* and *A. brassicicola* or other plant pathogens may reveal a phenotype for the *atnrx1* mutant.

Thiol-based regulation, based on the cellular redox status, is a sensitive mechanism employed by plants to cope with environmental fluctuations and biotic/abiotic stresses. Multifaceted redox proteins, such as AtNRX1, link external stimuli with phytohormone-mediated intracellular signaling networks to integrate diverse inputs and produce specifically tuned outputs that control plant defense, as well as growth and development [37,57]. These data advance our understanding of how redox regulation is integrated with other signaling modules to control various plant physiological states.

## 4. Materials and Methods

### 4.1. Materials

Chemicals including DTT, H_2_O_2_, MeJA, COR, and GSH were purchased from Sigma-Aldrich (St. Louis, MO, USA). Molecular markers for SDS-PAGE were purchased from Amersham Biosciences (Uppsala, Sweden). Anti-GFP, anti-HA, anti-MYC, anti-GST, and anti-6×His antibodies were obtained from Abcam (Cambridge, UK). Elicitor peptide 1 (PEP1) recombinant protein was purchased from MyBioSource (San Diego, CA, USA).

### 4.2. Plant Materials and Growth Conditions

*Arabidopsis thaliana* ecotype Columbia (Col-0) was used as the WT in this study. A homozygous T-DNA insertion mutant line, *atnrx1* (SALK_113401; Col-0 background), was obtained from the *Arabidopsis* Biological Resource Center (ABRC; Ohio State University, Columbus, OH, USA). Seedlings were grown on Murashige and Skoog (MS) medium (pH 5.7) containing 3% (*w*/*v*) sucrose and 0.25% (*w*/*v*) Phytagel™ (Sigma-Aldrich). *Arabidopsis* and tobacco plants were grown in growth chambers at 22 °C and 28 °C, respectively, under a 16 h light/8 h dark photoperiod and 100 μmol m^−2^ s^−1^ light intensity. JA and MeJA were dissolved in dimethyl sulfoxide (DMSO) to prepare 200 mM stock solutions of each.

### 4.3. Construction of Plasmids and Transgenic Plants

The full-length coding sequence (CDS) of *AtNRX1* was PCR-amplified from cDNAs using the primers AtNRX1-attB1_F and AtNRX1-attB2_R. The PCR product was extracted from the gel and used for secondary PCR amplification using the primers attB1_F and attB2_R. The DNA fragments amplified by the second PCR were cloned into Gateway entry vector *pDONR221* using the BP reaction kit, according to the manufacturer’s instructions (Invitrogen, Carlsbad, CA, USA), to generate the *pDONR-AtNRX1* construct. To generate Cys-to-Ser substitutions in AtNRX1M1, AtNRX1M2, and AtNRX1M3 proteins, the nucleotide sequence of *AtNRX1* was mutated using the QuikChange Site-Directed Mutagenesis Kit (Stratagene, La Jolla, CA, USA) with the following primer pairs: AtNRX1-C55,58S_F/_R for AtNRX1M2 and AtNRX1-C375,378S_F/_R for AtNRX2. The *AtNRX1* and *AtNRX1M3* sequences were cloned to the *pMDC43* vector [58] to generate the *pMDC43-AtNRX1* and *pMDC43-AtNRX1M3* constructs, respectively, which were then transformed into *Agrobacterium tumefaciens* strain GV3101 and further into *Arabidopsis* plants using standard protocols [59], thus generating transgenic *AtNRX1^OE^* and *AtNRX1M3^OE^* lines, respectively, using the *atnrx1* mutant. Transgenic lines were selected on medium containing 30 mg/L hygromycin and confirmed by genomic DNA-based PCR and sqRT-PCR.

### 4.4. Phenotypic Analysis of the Knockout Mutant and Overexpression Lines of Arabidopsis

To evaluate the role of AtNRX1 in resistance against fungal pathogens, 6-week-old WT, *atnrx1*, *AtNRX1^OE^*, and *AtNRX1M3^OE^* plants were inoculated with *Alternaria brassicicola* strain MUCL 20297 [60]. *A. brassicicola* was cultured on potato dextrose agar (PDA) medium (Difco, Sparks, MD, USA) at 28 °C for 2 weeks, and the spores were suspended in 0.02% Tween-20 for plant inoculation [30]. A 5 μL aliquot of the fungal spore suspension (5 × 10^5^ spores/mL) was dispensed dropwise onto the surface of plant leaves [61,62,63]. Disease symptoms and fungal growth were monitored at 3–7 dpi.

### 4.5. Expression of Recombinant Proteins

Full-length *AtNRX1* and *AtNRX1M1–3* CDSs were PCR-amplified using sequence-specific primers and cloned into the *pGEM-T Easy* vector (Promega, Madison, WI, USA). Then, *AtNRX1* and *AtNRX1M1–3* CDSs were excised out using *Bam*HI and *Eco*RI restriction endonucleases, and fragments were subcloned into *pGEX-2T* (Promega) and *pET28a* (Novagen, Madison, WI, USA) expression vectors. The resultant plasmids were transformed into *E. coli* BL21 (DE3; pLysS) for protein expression, as described previously [53]. The transformed cells were cultured at 37 °C in LB medium containing 50 μg/mL ampicillin until the absorbance of the culture solution at 600 nm (Abs_600_) reached 1.0. Glutathione-S-transferase (GST)-fused and 6×His-tagged proteins were purified using GST Sepharose 4 Fast Flow (GE Healthcare, Piscataway, NJ, USA) and Ni^2+^-charged IMAC-sepharose 6 Fast Flow (GE Healthcare), respectively. The GST and 6×His moieties were removed by cleavage with thrombin (GE Healthcare). AtNRX1 proteins were further purified using a TSK heparin-5PW high performance liquid chromatography (HPLC) column (7.5 × 75 mm). The purified AtNRX1 proteins were dialyzed against 20 mM HEPES-NaOH buffer (pH 7.5) for biochemical analysis.

### 4.6. Total RNA Isolation and Gene Expression Analysis

To analyze the expression of *AtNRX1* under stress conditions, *Arabidopsis* plants grown on 1× MS medium for 14 days were exposed to various abiotic stresses including high temperature (37 °C for 2 h), low temperature (4 °C for 6 h), dehydration (air-dried for 12 h), salt (300 mM NaCl), and an elicitor peptide (PEP1). Additionally, 6-week-old plants were challenged with necrotrophic fungal pathogens (*Botrytis cinerea* and *A. brassicicola*). Furthermore, to determine whether AtNRX1 is involved in the signaling of a specific plant hormone, the expression of the corresponding gene was analyzed in plants treated with 10 µM JA, 10 µM ETP, 10 µM ABA, 1 µM IAA, 1 µM GA, 10 nM eBL, and 20 µM zeatin. To analyze time-dependent *AtNRX1* expression, plants treated with *A. brassicicola* or MeJA were sampled at various time-points. The collected samples were immediately frozen in liquid N_2_ and used for RNA extraction. Total RNA was extracted from each sample using an RNA Extraction Kit (Qiagen, Valencia, CA, USA), and RNA concentration and purity were measured using a NanoDrop ND-1000 spectrophotometer (NanoDrop Technologies, Wilmington, DE, USA). To remove genomic DNA contamination, 1 μg total RNA was treated with RNase-free DNase I. The first strand of cDNA was synthesized using oligo(dT)_18_ primer and Revert Aid M-MuLV Reverse Transcriptase (Thermo Scientific, Rockford, IL, USA), according to the manufacturer’s instructions. Then, quantitative real-time PCR (qRT-PCR) was performed with the CFX 384 Touch^TM^ RT-PCR Detection System (Bio-Rad, Hercules, CA, USA) using the TOP real^TM^ qPCR 2X Pre MIX (SYBR Green with high ROX) Kit (Enzyomics, Daejeon, Korea), according to the manufacturer’s protocol. PCR cycling conditions were as follows: 95 °C for 15 min, followed by 40 cycles of 95 °C for 10 s, 55 °C for 10 s, and 72 °C for 30 s, and lastly a melting curve step to confirm the specificity of the amplified products. Three biological replicates were performed for each sample, and expression levels were normalized against *ACT2*, *UBQ1*, and *UBQ10* genes. Then, sqRT-PCR was performed using sequence-specific primer sets (Table S1). RT-PCR reactions were set up using 1 μL of 2-fold diluted cDNA (template) and 5 μM primers. PCR cycling conditions were as follows: 95 °C for 5 min, followed by 28 cycles of 95 °C for 30 s, 55 °C for 20 s, and 72 °C for 60 s. *ACT2* or *Tubulin2* (*TUB2*) served as a control.

### 4.7. Analysis of AtNRX1 Promoter Activity

The promoter region of *AtNRX1* (P_AtNRX1_; ~1 kb) was amplified from the genomic DNA of Col-0 plants by high-fidelity PCR using the primer pair P_AtNRX1_F-attB1/P_ANRX1_R-attB2. The resulting PCR product was cloned into Gateway *pDONR221* entry vector to produce *pDONR-P_AtNRX1_* using the BP reaction kit (Invitrogen), according to the manufacturer’s instructions. The P_AtNRX1_ sequence was excised from *pDONR-P_AtNRX1_* using *Nde*I restriction endonuclease, blunt-ended with DNA Polymerase I Large (Klenow) Fragment (New England Biolabs, Inc., Beverly, MA, USA), and then digested with *Bam*HI. The *35S* promoter was excised from the *pCAMBIA1305.1* binary vector (P_35S_) using *Bam*HI, blunt-ended with DNA Polymerase I Large Fragment, and then digested with *Bgl*II. Then, *P_AtNRX1_* was fused with the *GUS* reporter gene in the binary vector to generate the *P_AtNRX1_:GUS* construct, which was transformed into *A. tumefaciens* strain GV3101 and further into *Arabidopsis* plants using the floral dip method [59]. Transformed seeds were selected on MS agar medium supplemented with 10 μg mL^−1^ hygromycin (Duchefa, Haarlem, The Netherlands) and/or 250 μg mL^−1^ cefotaxime (Duchefa). Fourteen-day-old seedlings were subjected to mock (DMSO) or MeJA treatment for 36 h, and then whole seedlings were used for GUS staining assays, as described previously [64]. In brief, seedlings were immersed in the GUS staining solution (1 mg/mL 5-bromo-4-chloro-3-indolyl-β-D-glucuronide, 200 mM sodium phosphate [pH 7.0], 0.5 mM ferricyanide, 0.5 mM ferrocyanide, and 10 mM DTT) and then incubated overnight at 37 °C in the dark. Tissues were cleared using 70% ethanol, and GUS images were taken using a digital camera.

### 4.8. Redox-Dependent Structural Switching of AtNRX1

The redox-dependent structural modification of AtNRX1 was analyzed in vitro by treating the purified recombinant AtNRX1 protein with 10 mM DTT or 10 mM H_2_O_2_. To trace redox-dependent structural changes in AtNRX1 in planta, *A. tumefaciens* strains carrying the *GFP-AtNRX1* or *GFP-AtNRX1M3* construct were infiltrated to tobacco leaves treated with or without X/XO (100 mM/0.05 U mL^−1^) and 10 mM GSH for 3 h. Leaf samples were harvested at 3 dpi and ground in liquid N_2_. Total protein extracts were separated on 10% reducing or non-reducing acrylamide gels by SDS-PAGE and subjected to western blotting with anti-GFP antibody.

## Supplementary Materials

The following are available online at https://www.mdpi.com/1422-0067/21/23/9196/s1.

## Abbreviations

SDS-PAGE	Sodium dodecyl sulfate-polyacrylamide gel electrophoresis
GFP	Green fluorescent protein
GSGST	Glutathione-S-transferase
HEPES	N-2-hydroxyethylpiperazine-N’-2-ethanesulfonic acid
GUS	β-glucuronidase
TRX	thioredoxin
NRXs	nucleoredoxins
qPCR	Quantitative polymerase chain reaction
sqRT-PCR	semi-quantitative reverse transcription PCR
HPLC	high performance liquid chromatography
*AtNRX1*	*ARABIDOPSIS NUCLEOREDOXIN 1*
*ACT2*	*Actin2*
*TUB2*	*Tubulin2*
*UBQ1*	*Ubiquitin1*
*PDF1.2*	*PLANT DEFENSIN 1.2*
*AbCutA*	*A. brassicicola Cutinase A*
Dvl1	Disheveled 1
JA	jasmonic acid
SA	salicylic acid
MeJA	methyl jasmonate
ETP	ethephon
ABA	abscisic acid
IAA	indole-3-acetic acid
GA_3_	gibberellin A_3_
eBL	24-epibrassinolide
OPDA	cis-12-oxophytodienoic acid
PDA	potato dextrose agar
LMW	low molecular weight
TFs	transcription factors
ROS	Reactive oxygen species
DTNB	5,5-dithio-bis-(2-nitrobenzoic acid)
TR	thioredoxin reductase
DTT	1,4-dithiothreitol
GPx	glutathione peroxidase
NPR1	non-expressor of PR1
X/XO	xanthine/xanthine oxidase
NLS	nuclear localization signal
NES	nuclear export signal
TGA	TGACG-motif binding
JAZ	JASMONATE ZIM-DOMAIN
