# Supplementary material for: Redox-Dependent Structural Modification of Nucleoredoxin Triggers Defense Responses against Alternaria brassicicola in Arabidopsis
Source: Int J Mol Sci. 2020 Dec 2;21(23):9196. doi: 10.3390/ijms21239196 (PMC7730559; doi:10.3390/ijms21239196)
Supplement: Supplementary file 1 [file ijms-21-09196-s001.pdf]

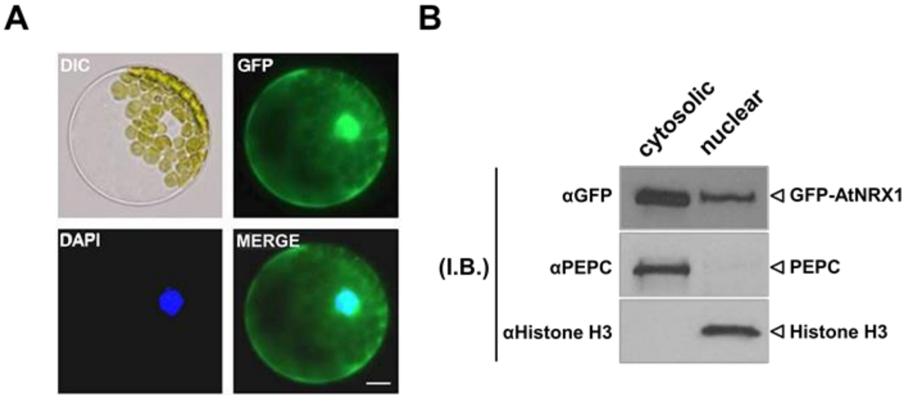

Figure S1. Subcellular localization of AtNRX1 in *Arabidopsis*. (A) Transient expression of *GFP-AtNRX1* in *Arabidopsis* protoplasts. Protoplasts were transformed with the *GFP-AtNRX1* construct (depicted in Figure S1B), and localization of the fusion proteins was examined after 48 h by confocal laser-scanning microscopy. At least three independent transformation experiments were carried out, and representative images are shown. The localization of GFP-AtNRX1 to the nuclei was detected by 4′ ,6-diamidino-2-phenylindole (DAPI) staining. GFP: green fluorescence signal, which indicates the location of AtNRX1 in Arabidopsis protoplasts; DAPI: blue fluorescence signal, which indicates the location of the nucleus stained by DAPI; DIC: differential interference contrast (DIC) image; MERGE: the combined image of GFP and DAPI signals. Scale bar = 10 μm. (B) Localization pattern of GFP-AtNRX1 analyzed by fractionation analysis. The *GFP-AtNRX1* construct was infiltrated into tobacco leaves, and total protein was extracted from leaves at 2 dpi. GFP-AtNRX1 was present in both cytoplasmic and nuclear fractions. The level of GFP-AtNRX1 protein was determined by immunoblotting (I.B.) with anti-GFP antibody. The degree of enrichment was determined using antibodies against marker proteins (anti-histone H3, nuclear marker; anti-PEPC, cytosolic marker). Experiments were repeated three times with similar results.

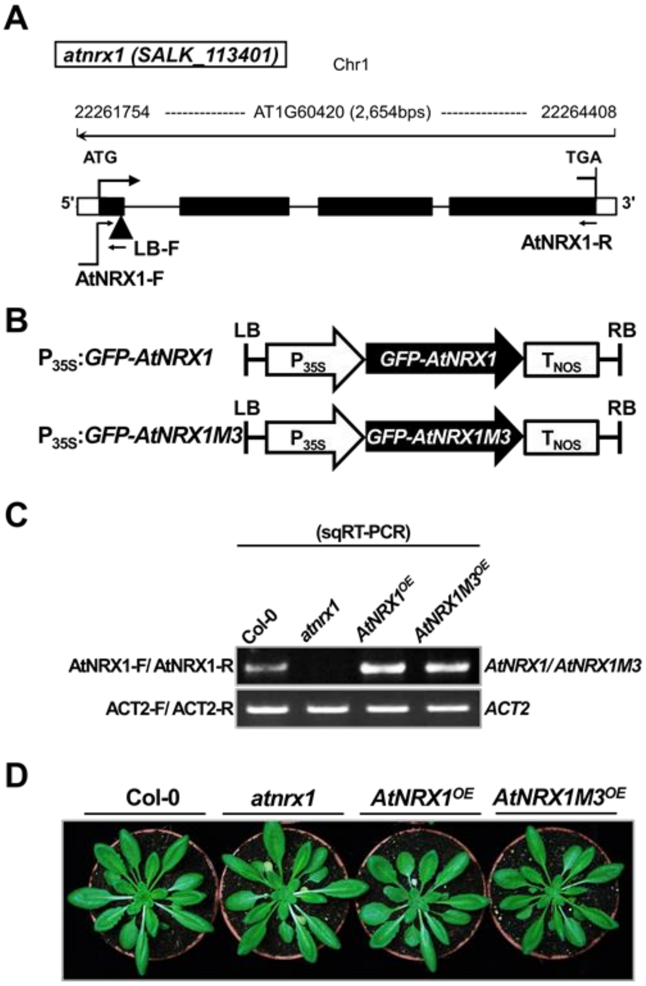

Figure S2. Confirmation of T-DNA mutant and overexpression lines used in this study. (A) Schematic representation of the predicted structure of *atnrx1*. The black elbow arrow represents the start codon ATG; filled boxes indicate exons; a horizontal black line indicates introns; empty boxes at both ends represent 5′ and 3′ untranslated regions (UTRs). The position of the T-DNA insertion is indicated by a black arrowhead. Black arrows represent the primer positions used for sqRT-PCR in (C). (B) Schematic representation of the constructs used to generate *AtNRX1*^{OE} (P_{35S}*:GFP-AtNRX1*) and *AtNRX1M3*^{OE} (P_{35S}*:GFP-AtNRX1M3*) lines. P_{35S} and T_{NOS} represent the CaMV *35S* promoter and *nopaline synthase* (*NOS*) terminator, respectively. (C) Identification of homozygous mutant and OE plants in comparison with the WT (Col-0). The indicated primer pairs were used for sqRT-PCR analyses. *ACT2* was used as a control. (D) A photo showing 3-week-old WT (Col-0), *atnrx1*, *AtNRX1*^{OE}, and *AtNRX1M3*^{OE} plants grown under long-day photoperiod.

Table S1. List of PCR primers used in this study.

Gene	AGI code	Oligo name	Sequence (5'-3')	Purpose
AtNRX1	AT1G60420	AtNRX1-qRT_F	CCGGCGAGGGATTTCC	
		AtNRX1-qRT_R	CTTCCCTAACAAGCTGTCAACTTTC	
PDF1.2	AT5G44420	PDF1.2-qRT_F	GCATTAACCTTGAAGGAGCC	
		PDF1.2-qRT_R	GTTACTCATAGAGTGACAGAG	
AbCutA	†P41744	AbCutA-qRT_F	CACTGCGCCCAATGATGAAC	
(CUTAB1)		AbCutA-qRT_R	GTAGCCGAACAACACGACACC	**qRT**
ACT2	AT3G18780	ACT2_qF	TGATGCACTTGTGTGTGACAA	**-PCR**
		ACT2_qR	GGGACTAAAACGCAAAACGA	
UBQ1	AT3g52590	UBQ1_qF	TTCCTTGATGATGCTTGCTC	
		UBQ1_qR	TTGACAGCTCTTGGGTGAAG	
UBQ10	AT4G05320	UBQ10_qF	AGATCCAGGACAAGGAGGTATTC	
		UBQ10_qR	CGCAGGACCAAGTGAAGAGTAG	
PDF1.2	AT5G44420	PDF1.2-qRT_F	ATGGCTAAGTTTGCTTCCAT	
		PDF1.2-qRT_R	TTAACATGGGACGTAACAGA	
TUB2	AT5G62690	TUB2sqRT_F	ATGCGTGAGA TTCTTCACAT	**sqRT**
		TUB2sqRT_F	TCAGTACTCTTCCTCCTGTT	**-PCR**
ACT2	AT3G18780	ACT2_sqF	ATGGCTGAGGCTGATGATAT	
		ACT2_sqR	TTAGAAACATTTTCTGTGAACG	
AtNRX1	AT1G60420	AtNRX1_F	ATGGCCGAAACCTCGAAGCA	
		AtNRX1_R	TCAGGCCTTGGTGCATACGT	
TUB2	AT5G62690	TUB2_F	CCAACAACGTGAAATCGACA	**Geno**
		TUB2_R	TCTTGGTATTGCTGGTACTC	**-typing**
Lba-T-DNA	†	LB_F	TGGTTCACGTAGTGGGCCATCG	
NOS	†	NOS_R	AGTAACATAGATGACACCGCGCGCGAT	
AtNRX1	AT1G60420	AtNRX1-attB1_F	AAAAAGCAGGCCATATGGCCGAAACCTCGAAGCA	
		AtNRX1-attB2_R	AGAAAGCTGGGTTCAGGCCTTGGTGCATACGT	
		AtNRX1(NoEND)-attB2_R	AGAAAGCTGGGTGGCCTTGGTGCATACGT	**Cloning**
††*attB site*	†	attB1	GGGGACAAGTTTGTACAAAAAAGCAGGCCAT	
		attB2	GGGGACCACTTTGTACAAGAAAGCTGGGT	
AtNRX1	AT1G60420	AtNRX1-C55,58S_F	GCTGCTTGGTcTGGACCGTcTCAGCGGTTT	
		AtNRX1-C55,58S_R	AAACCGCTGAgACGGTCCAgACCAAGCAGC	**Mutation**
		AtNRX1-C375,378S_F	GCTCACTGGTcTCCTCCTTcTCGCGCTTTT	
		AtNRX1-C375,378S_R	AAAAGCGCGAgAAGGAGGAgACCAGTGAGC	

† These genes do not have AGI codes. †† Reference: Craig NL, Nash HA. *E. coli* integration host factor binds to specific sites in DNA. Cell. 1984 Dec;39(3 Pt 2):707-16.
